# Machine Learning-Based Classification of Soil Parent Materials Using Elemental Concentration and Vis-NIR Data

**DOI:** 10.3390/s24165126

**Published:** 2024-08-07

**Authors:** Yüsra İnci, Ali Volkan Bilgili, Recep Gündoğan, Gafur Gözükara, Kerim Karadağ, Mehmet Emin Tenekeci

**Affiliations:** 1 Organized Industrial Zone Vocational School, Harran University, Sanliurfa 63300, Türkiye; inci@harran.edu.tr; 2 Department of Soil Science and Plant Nutrition, Faculty of Agriculture, Harran University, Sanliurfa 63300, Türkiye; rgundogan@harran.edu.tr; 3 Department of Soil Science and Plant Nutrition, Eskisehir Osmangazi University, Eskisehir 26160, Türkiye; ggozukara@ogu.edu.tr; 4 Department of Soil Science, University of Wisconsin-Madison, Madison, WI 53706, USA; 5 Department of Electrical and Electronics, Faculty of Engineering, Harran University, Sanliurfa 63300, Türkiye; k.karadag@harran.edu.tr; 6 Department of Computer Science, Faculty of Engineering, Harran University, Sanliurfa 63300, Türkiye; etenekeci@harran.edu.tr

**Keywords:** soil science, classification, XRF, Vis-NIR, ICP-OES

## Abstract

In soil science, the allocation of soil samples to their respective origins holds paramount significance, as it serves as a crucial investigative tool. In recent times, with the increasing use of proximal sensing and advancements in machine-learning techniques, new approaches have accompanied these developments, enhancing the effectiveness of soil utilization in soil science. This study investigates soil classification based on four parent materials. For this purpose, a total of 59 soil samples were collected from 12 profiles and the vicinity of each profile at a depth of 0–30 cm. Surface soil samples were analyzed for elemental concentrations using X-Ray fluorescence (XRF) and inductively coupled plasma–optical emission spectrometry (ICP-OES) and soil spectra using a visible near-infrared (Vis-NIR) spectrometer. Soil samples collected from soil profiles (12 soil samples) and surface (47 soil samples) were used to classify parent materials using machine learning-based algorithms such as Support Vector Machine (SVM), Ensemble Subspace k-Near Neighbor (ESKNN), and Ensemble Bagged Trees (EBTs). Additionally, as a validation of the classification techniques, the dataset was subjected to five-fold cross-validation and independent sample set splitting (80% calibration and 20% validation). Evaluation metrics such as accuracy, F score, and G mean were used to evaluate prediction performance. Depending on the dataset and algorithm used, the classification success rates varied between 70% and 100%. Overall, the ESKNN (99%) produced better results than other classification methods. Additionally, Relief algorithms were employed to identify key variables for each dataset (ICP-OES: CaO, Fe_2_O_3_, Al_2_O_3_, MgO, and MnO; XRF: SiO_2_, CaO, Fe_2_O_3_, Al_2_O, and MnO; Vis-NIR: 567, 571, 572, 573, and 574 nm). Subsequent soil reclassification using these reduced variables revealed reduced accuracies using Vis-NIR data, with ESKNN still yielding the best results.

## 1. Introduction

Soil classification is crucial not just for agriculture but also for a range of applications, such as forensic science, engineering, and land-use management [1,2,3,4]. Soils with a highly diverse and complex structure vary in their mineralogical and elemental compositions depending on factors such as soil formation processes, the parent material (rocks), climate, topography, and land use [1,5,6]. Understanding the origins and properties of soils is crucial in soil classification studies [2]. Various analytical methods, such as color analysis, particle size distribution analysis, pollen analysis, and magnetic susceptibility, have been employed to characterize soils [1,7]. Also, techniques such as scanning electron microscopy (SEM), atomic absorption emission spectrometry (AAS), inductively coupled plasma–mass spectrometry (ICP-MS), or inductively coupled plasma–optical emission spectrometry (ICP-OES) were utilized to determine the quantities of inorganic materials and minerals [2,5,6,8]. However, these approaches, especially those involving chemical usage, can be expensive and time-consuming [6].

Recently, the increased utilization of proximal sensing and advancements in machine-learning techniques have led to new approaches, enhancing the effectiveness of soil characterization in soil science. Techniques such as X-ray fluorescence (XRF), Vis-NIR, mid-infrared (MIR), and X-ray diffraction (XRD) are commonly employed for characterizing soils using proximal sensing methods in this field [9].

XRF spectrometry provides a rapid and eco-friendly means of determining total elemental concentrations in soils without generating hazardous waste. This technology has proven reliable, non-destructive, fast, and cost-effective for soil characterization. This technology relies on the emission of secondary X-rays from soil material when excited by high-energy X-rays, enabling the calculation of diverse elemental compositions [10,11,12]. The XRF technique is employed to determine the chemical characteristics of soils, offering insights into the parent material, weathering conditions, and intensity of pedogenic processes that influence soil formation [11].

Instrumental techniques like XRF and ICP-OES, in addition to determining total element concentrations in soils, have been employed for various purposes, including predicting soil properties, assessing soil fertility and plant productivity, examining soil development, analyzing soil pollution, and evaluating the history of soil formation processes and weathering [11,12,13,14,15]. Moreover, the combination of XRF with Vis-NIR spectral data has been utilized for predicting specific soil productivity and physicochemical properties [11,16]. The effective use of the XRF technique, providing information about the elemental concentrations of soils, has proven beneficial in assessing the origin of soil parent materials [14].

The collection of reflections from Vis-NIR regions of soil surfaces has been employed to distinguish soils based on their origins [17,18,19,20]. This method offers advantages such as no sample preparation requirements, reduced chemical usage, the ability to analyze a large number of samples, and cost-effectiveness [18,19]. Soil classification based on spectral properties involves examining the shapes of spectral curves, correlating with diverse physicochemical properties of the soils [19]. Variations in clay and sand ratios, organic matter content, iron oxide ratios, and mineral composition result in soils exhibiting reflections of differing intensities or possessing absorption peaks at distinct wavelengths. An increase in clay content or high levels of organic matter and Fe_2_O_3_ ratios lead to lower reflection intensity, while soils containing quartz-type minerals and having a high sand content tend to display higher reflectance [17].

Understanding the parent material is crucial, given that factors such as topography and parent material significantly influence soil-formation processes. This knowledge can provide valuable insights into the origins of soils [14].

While XRF, ICP-OES, and Vis-NIR methods have been separately assessed for soil classification, there is a shortage of studies concurrently analyzing their classification success on identical samples using different machine-learning methods. To address this gap, the current study attempted to employ various proximal sensing techniques to unravel the origins of soil samples. To accomplish this, three distinct profiles were established on four different parent materials (12 soil profiles in total), with a variable number of surface samples collected from each profile. Utilizing XRF, ICP-OES, and Vis-NIR techniques, alongside diverse machine-learning approaches, like SVM, EBT, and ESKNN, the study aims to provide comprehensive insights into soil science.

## 2. Materials and Methodology

### 2.1. Study Area and Soil Sampling

The study area covers the province of Sanliurfa. Sanliurfa province, which is located in the Southeastern Anatolia region of Turkey, is approximately 200 thousand km^2^ and is located between 36,038′00″–37,059′37″ northern latitudes and 37,049′03″–40,014′37″ east longitudes. Sanliurfa province is of pivotal significance, as it is a key component of the GAP (Southeastern Anatolia Project), one of the world’s largest irrigation and electricity production initiatives. This project aims to irrigate an expansive 1.8 million hectares of land, making it a crucial contributor to agriculture and energy production in the region. Moreover, with the construction of the fourth largest dam globally, Sanliurfa has become pivotal in facilitating enhanced agricultural capabilities and attracting significant migration flows seeking economic opportunities [21]. Sanliurfa province is in an arid and semi-arid climate zone, and the soil temperature and moisture regimes are classified as xeric and thermic. The summers are hot and dry, and the winters are mild and rainy. The mean temperature and precipitation are 17.2 °C and 451 mm.

**Figure 1:**
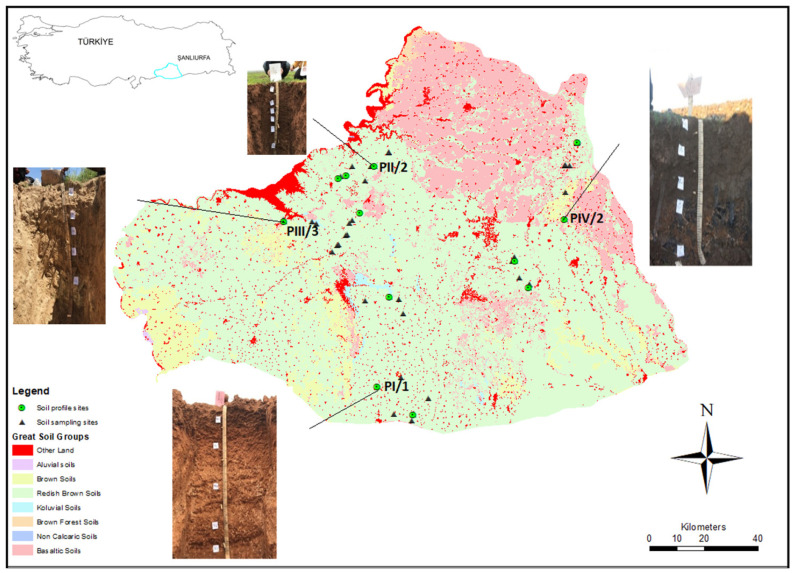
Study area (Sanliurfa), profiles, and soil-sampling locations.

**Figure 2:**
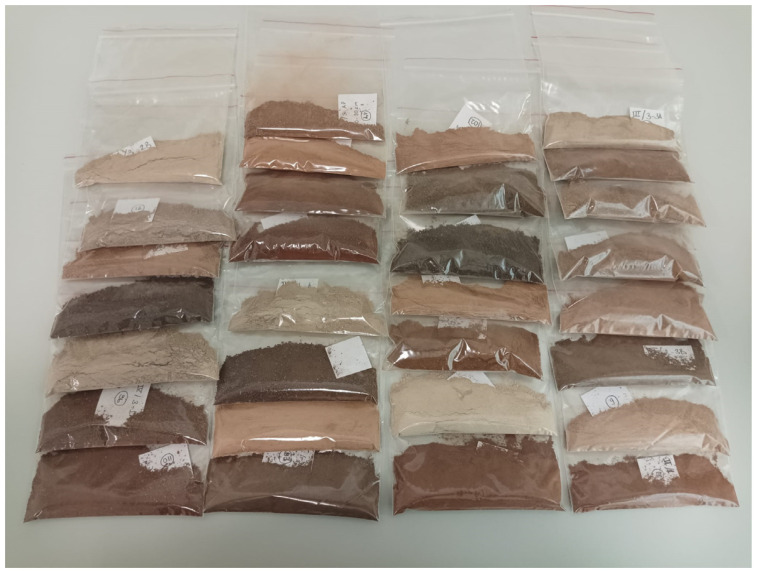
Soil samples from various parent materials studied and color differences observed: dark brown (basalt), dark reddish brown (limestone), reddish brown (mudflow), and light reddish brown (Marn).

Three different profiles were opened on four different parent materials (I—Mud flows; II—Eocene–Miocene limestones; III—Neogene marls; and IV—Basalt materials), resulting in a total of 12 soil profiles. Each profile was sampled horizon by horizon. Additionally, soil samples were randomly taken from the surface around each profile. In this study, surface horizon samples from each horizon, along with randomly collected surface samples around the profiles, were used, totaling 59 samples (Figure 1 and Figure 2). The elevations of the soil profile sampling locations ranged from 368 to 1424 m above sea level. The soils were classified according to the soil taxonomy system as Vertisols and Inceptisols orders [22]. The soils of the study exhibit varying characteristics depending on different parent materials, characterized by a high clay content, low organic matter, and elevated calcium content influenced by the properties of dry areas [23]. The collected soil samples were dried under room temperature and sieved using a 2 mm sieve for ICP-OES, XRF, and Vis-NIR spectral reflectance. Detailed information about soil profiles can be found in Table 1.

### 2.2. Soil Instrumental and Spectroradiometric Analyses

#### 2.2.1. ICP Analyses

To determine the concentrations of total elemental components (Si, Al, Ca, Fe, Mg, K, P, and Mn) in the samples using the inductively coupled plasma–optical emission spectroscopy (ICP-OES) technique, compounds present in the soil samples were initially dissolved into a solution. To achieve this, a sufficient number of soil samples were ground (to a particle size of 150 µm or less) and then subjected to drying in an oven at temperatures at 105 °C to eliminate moisture. Moisture removal was monitored by weighing the samples periodically until a constant weight was reached, indicating complete moisture removal. Once the drying process was completed, 1 g of the finely ground dried soil sample was transferred to microwave digestion vessels. To this, 6.5 mL of HNO_3_ and 3.5 mL of HCl with purities of 65% and 37%, respectively, were added to the samples. After approximately 5 min, allowing gases produced by the reaction of acids with organic compounds to dissipate, the vessels were sealed. The microwave oven was programmed appropriately, and the process was initiated. Digestion was continued until no residue remained, ensuring that the soil was completely dissolved into the solution. The obtained solution was then diluted to specific volumes (e.g., 50 or 100 mL). A portion of this solution was taken for the desired ICP-OES analyses. To calibrate the equipment, standards (Merc^®^, Darmstadt, Germany) prepared at various concentrations spanning the range of the samples are utilized. Additionally, to confirm the reliability of the results, the equipment is validated using samples of known concentrations. ICP-OES analyses were conducted using the ICP-OES Optima 5300 DV instrument (PerkinElmer Life and Analytical Sciences, Shelton, CT, USA). The microwave digestion system used for sample digestion was the Berghof speed wave MWS-2 (Berghof Products + Instruments GmbH, Eningen unter Achalm, Germany). The working conditions for the ICP-OES apparatus are outlined in Table 2.

#### 2.2.2. XRF Analyses

To determine the concentrations of Si, Al, Ca, Fe, Mg, K, P, and Mn using the XRF technique, approximately 40–50 g of soil powder (ground to a particle size of 150 µm or less) was initially taken. The samples were left only 4–5 h at 105 °C for drying in the oven without waiting too long; thus, it is thought not to damage the soil samples due to low organic matter content, which mitigates potential damage to the soil samples. Following drying, 4 g of the dried soil sample was combined with 0.9 g of binder material (Wachs) and pressed into pellets, using a hydraulic press, for analysis. Once prepared, the major elemental (Si, Al, Ca, Fe, Mg, K, P, and Mn) concentrations of the samples were determined utilizing the XRF device at the Ankara University Earth Sciences Application and Research Center (YEBİM). The analyses were performed on a Spectro brand X-LAB 2000 model Polarized Energy Dispersive X-Ray Fluorescence Spectrometer (PEDXRF) (Spectro Analytical Instruments GmbH, Kleve, Germany) device for main element oxide and trace element analyses.

#### 2.2.3. Vis-NIR Analyses

Approximately 20 g of each air-dried and sieved soil sample was placed in glass petri dishes to prepare the samples for spectral scanning. In the laboratory, the soil samples’ reflections in the wavelength range of 350–2500 nm were obtained with a spectral resolution of 1 nm using a Vis-NIR spectroradiometer device ASD FieldSpecPro 3 (Malvern Panalytical Ltd., Malvern, UK). The final reflections of the soils (*R*) were obtained by ratioing the reflections obtained from the soil samples to those obtained from the white standard (white Spectralon standard material):

$$R=\frac{Soil\;Reflection}{White\;Spectralon\;Reflection\;(Referans)}$$

White Spectralon was used at regular intervals for the calibration of the Vis-NIR spectroradiometer.

In this study, only raw spectra were used without any preprocessing. Both in this study and in previous research, no significant improvement was observed when preprocessing the data, such as applying the first derivative to the raw spectra.

### 2.3. Feature Selection with Relief Method

The Relief method is applied in classification procedures to assess the significance of features in differentiating classes. The algorithm imposes penalties on attributes that provide varying values for neighbors within the same class, while rewarding attributes that yield different values for neighbors from distinct classes. This process calculates the weight assigned to each attribute. Initially, all attributes are set to a value of 0. Subsequently, a random sample (*x_r_*) is chosen, and the relevance of features (*F_j_*) is updated based on the k-nearest neighbor sample (*x_q_*) relative to the selected sample. This iterative process is applied to all features, resulting in the computation of feature weights. Computations are performed based on formula in Equation (1).

(1) $$W_{j}^{i}=\left\{\begin{array}{c} W_{j}^{i}=W_{j}^{i-1}-\frac{\Delta \left(x_{r},x_{q}\right)}{m}\cdot d_{rq}\;,x_{r}\;and\;x_{q}\;same\;class\;\\ W_{j}^{i}=W_{j}^{i-1}+\frac{P_{y_{q}}}{1-P_{y_{r}}}\cdot \frac{\Delta \left(x_{r},x_{q}\right)}{m}\cdot d_{rq},\;x_{r}\;and\;x_{q}\;different\;class \end{array}\right.$$

where *W* represents the weight of attributes; $P_{y_{r}}$ is probability of the class to which $x_{r}$ belongs, $P_{y_{q}}$; $x_{q}$ is the probability of the sample in a different class; *m* is the number of iterations; $\Delta \left(x_{r},x_{q}\right)$ is used to calculate the difference between samples for the *F_j_* attribute, as shown in Equation (2); and $x_{rj}$ represents the *j*th attribute of $x_{r}$.

(2) $$\Delta \left(x_{r},x_{q}\right)=\frac{\left|x_{rj}-x_{qj}\right|}{\max\left(F_{j}\right)-min(F_{j})}$$

where $d_{rq}$ calculates the value as the distance between samples, as shown in Equation (3); $rank\left(r,q\right)$ determines the order of the *r* sample according to *q* neighbor; and *k* indicates how many neighbors will be examined. Sigma value is used for scaling.

(3) $$d_{rq}=\frac{e^{-{\left(\frac{rank\left(r,q\right)}{sigma}\right)}^{2}}}{\sum_{l=1}^{k}e^{-{\left(\frac{rank\left(r,q\right)}{sigma}\right)}^{2}}}$$

### 2.4. Classification

**Figure 3:**
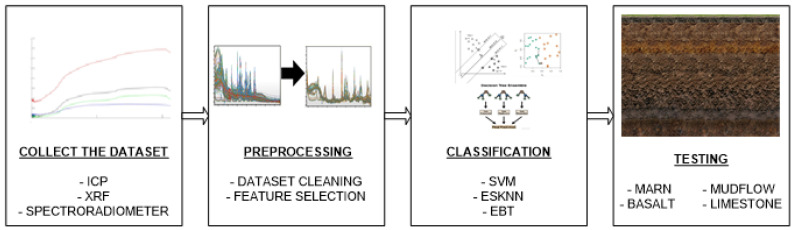
Steps followed in the classification of soils from different parent materials.

The classification of soils based on different parent materials (Mudflow, Limestone, Marn, and Basaltic), using XRF, ICP, and spectroradiometric data along with three distinct machine-learning algorithms—Support Vector Machine (SVM), Ensemble Subspace k-Near Neighbor (ESKNN), and Ensemble Bagged Trees (EBT)—is outlined in Figure 3. Following the acquisition of instrumental and spectroradiometric data, the effectiveness of classification models, developed using various machine-learning techniques and datasets, was evaluated through three different approaches. (1) Surface samples from profiles opened on four different parent materials were used in the calibration set, while soil samples collected from around the profiles were used in the validation set. (2) The classification results were validated using tenfold cross-validation. (3) The entire dataset was randomly divided into two parts: calibration (80%) and validation (20%) datasets. Additionally, (4) classifications were repeated by selecting important elements obtained by ICP-OES and XRF, and important wavelengths in the Vis-NIR dataset, using the Relief algorithm.

#### 2.4.1. Methods Used in Classification

For the classification process, cubic SVM, ESKNN, and EBT methods were used. Classifications with different methods were run with three different inputs, which are ICP-OES, XRF, and Vis-NIR data, and also significant elements and raw reflection values at important wavelengths determined by the Relief algorithm.

SVM is a form of supervised learning algorithm utilized for binary classification and regression tasks. This technique constructs an optimal hyperplane to serve as the decision boundary, which maximizes the margin of separation between the two classes within the dataset. The training of an SVM involves two primary phases: firstly, the input data are mapped into a high-dimensional feature space. Subsequently, a quadratic optimization problem is solved to derive the optimal hyperplane that effectively discriminates between the two classes based on the transformed features.

ESKNN is an advanced variant of the traditional KNN algorithm, where multiple models or algorithms are employed in conjunction by reducing the dimensions or feature numbers in the dataset to determine the most optimal approach. KNN is a sample-based classification algorithm that is relatively straightforward to implement compared to other classification methods, and it does not require a training phase. In KNN, the classification of a new sample is performed using known samples from the dataset. Each sample to be classified is compared individually with every sample in the training set. To determine the class of the test sample, the algorithm selects the k-nearest neighbors within the training set based on their proximity to the test sample. The distance between samples is typically calculated using the Euclidean distance metric. The Euclidean equation used for distance measurement is given in Equation (4) [24]. By leveraging ensemble methods, ESKNN enhances the traditional KNN approach by integrating multiple models to improve classification accuracy and robustness. This ensemble approach mitigates some of the limitations inherent in KNN, such as sensitivity to the choice of k and vulnerability to the curse of dimensionality.

(4) $$\sqrt{\sum_{i=1}^{k}{\left(xi-yi\right)}^{2}}$$

The EBT allows for the training of multiple decision tree models, using the same algorithm, by creating different training datasets. A DT constructs a hierarchical structure of branches and nodes based on the feature vector set. Initially, a root node is selected to minimize impurity across its two child nodes, independent of the feature variable it originates from, as formulated in Equation (5):

(5) $$im\left(r\right)=-\sum_{i=1}^{m}p\left(w_{i}\backslash r\right)logp(w_{i}\backslash r)$$

In this context, *im*(*r*) denotes the impurity measure at node *r*, where *p*(*w_i_*|*r*) represents the proportion of patterns, *x_i_*, assigned to class *w_i_* within node *r*. Each non-terminal node is recursively split into two child nodes, *r*1 and *r*2, with *p*_1_ and *p*_2_ indicating the respective proportions of instances directed to these nodes. The most suitable split is determined by maximizing the difference described in Equation (6).

(6) $${∆}_{im}\left(d,r\right)=im\left(r\right)-p_{1}im\left(r1\right)-p_{2}im\left(r2\right)$$

The decision tree (DT) continues to grow until a stage is reached where further partitioning with an additional segment, d, does not significantly reduce the noise criterion. At this juncture, node *r* ceases further subdivision and transitions automatically into a terminal node. The class *w_i_*, associated with terminal node r, is determined by maximizing the conditional probability, p(winr). Test samples are classified using the optimized DT model derived from this process [25].

The performance of various classification methods was evaluated using 5-fold cross-validation. In this process, all variables are sequentially classified, and classification models are trained by iteratively excluding variables and predicting the discarded variable. This method ensures a robust evaluation of the classifiers’ abilities to generalize across different subsets of the data.

#### 2.4.2. Performance Evaluation Metrics

The performance and accuracy evaluations of SVM, ESKNN, and EBT classification methods were conducted using metrics such as accuracy (Acc), F-Score, and G-Mean, as defined in Equations (7)–(9).

(7) $$Acc=\frac{TP+TN}{TP+FP+TN+FN}$$

(8) $$\mathrm{F-Score}=2*\frac{\left(\frac{TP}{FP+TN}\right)*(\frac{TP}{TP+FN})}{\left(\frac{TP}{FP+TN}\right)+(\frac{TP}{TP+FN})}$$

(9) $$\mathrm{G\_Mean}=\sqrt{\left(\frac{TP}{TP+FN})+(\frac{TN}{FP+TN}\right)}$$

All analyses of machine-learning algorithms in the study were made on a computer with an operating system Windows 10 Pro with CPU (Intel Core i7-8565) 3.00 GHz, 8 GB RAM, and 1 TB hard disk. Machine learning (ML) algorithms were performed using a MATLAB R2018a software program.

## 3. Results and Discussion

### 3.1. Total Elements

The distribution of total element concentrations obtained through ICP-OES and XRF techniques is provided in Table 3. The highest percentage was observed for SiO_2_, followed by CaO, Al_2_O_3_, or Fe_2_O_3_, depending on the parent material, and also with the ranking varying according to the parent material. Other elements (MgO, K_2_O, P_2_O_5_, and MnO) followed these based on the parent material. In contrast, CaO ratios were found to be higher than SiO_2_ ratios in the Marl parent material. Overall, while exhibiting similarities to the element distributions in soils from previous studies [26,27,28], they also reflect the characteristics of soils in arid and semi-arid regions [10]. The concentrations of all elements were statistically significant among the parent materials, indicating their discriminative power among soils formed on different parent materials. The detailed information regarding the variation in element concentrations according to different materials can be found in the previous study [29]. As noted in a previous study, lower elemental concentrations in ICP analyses compared to XRF analyses were observed, primarily due to differences in the effectiveness of acids used during sample pretreatment to dissolve all components [29].

### 3.2. Spectral Features

**Figure 4:**
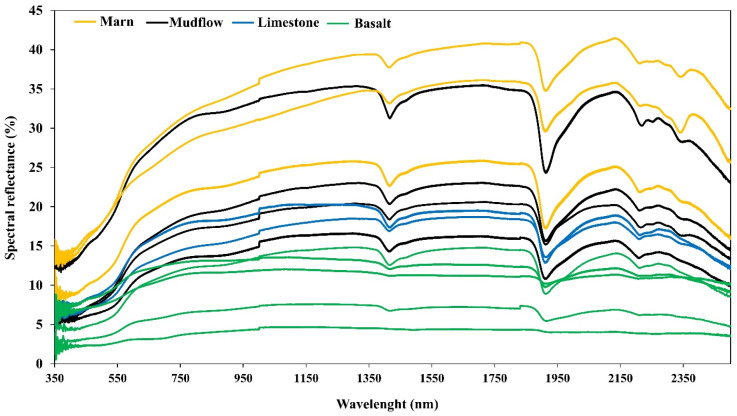
Average reflectances of surface samples of soils formed on different parent materials.

Graphs depicting the average reflectance values representing different parent materials were generated. The obtained graphs are presented in Figure 4. The percent reflectance levels of soils varied depending on the content of the soils. The spectral reflections obtained from soil samples formed on different parent materials using the Vis-NIR technique also exhibited variations in mean values according to the parent materials, similar to the total element contents. The lowest reflectance intensity was obtained from basaltic soils, and this low reflectance intensity is attributed to the richness of these soils in iron oxide (Table 2). Soils with higher Fe_2_O_3_ content tend to exhibit lower reflectance intensity [17]. Peaks in the 400 to 1000 nm range are generally associated with iron oxides, minerals such as hematite and goethite, and organic matter. Absorption bands between 1400 and 2200 nm are linked to hydroxyl groups and soil water, and, specifically within the range of 2100 to 2500 nm, they are connected with clay minerals like kaolinite, illite, and smectite. Notably, the band around 2350 nm is correlated with the high CaCO_3_ content in the soil [17,19,30].

### 3.3. Classification

The success of classifying soil samples into their sources (parent materials) using traditional ICP-OES and proximal sensing techniques (XRF and Vis-NIR), with the help of various machine-learning methods, has been tested with four different approaches. As a classification method, ESKNN, SVM, and EBT models were chosen. The results obtained using all features were processed by splitting the data into training-test sets at an 80–20% ratio and performing fivefold cross-validation, as well as utilizing profile-based training with surrounding validation examples. The metrics, including the F score, accuracy, and G mean, were employed to evaluate the effectiveness of a classification model in each distinct classification approach. These metrics provide insights into various aspects of the model’s performance, including precision, recall, overall correctness, and balance between true-positive and true-negative rates [31]. All error metrics used to evaluate the classification success of the algorithms (accuracy, F Score, and G_Mean) range between 0 and 1. The closer the values are to 1, the higher the classification performance or success of the models can be considered. The achieved success rates are provided in Table 4.

The classification successes varied based on the employed machine-learning technique, classification approach, and the analysis method used. The classification accuracies of models created using different inputs varied depending on the dataset and algorithm used, ranging between 0.7 and 1. Near-perfect classification accuracy, nearing 100%, was achieved. Among the inputs used, generally, the results from the Vis-NIR technique, as well as XRF results, provided classification outcomes that were both close to each other and more successful compared to the ICP technique. However, in more instances, Vis-NIR has been slightly more successful than XRF in terms of classification accuracy (Table 4). In studies where different inputs and similar machine-learning techniques or different machine-learning techniques are used, near 100% successful results have been achieved [19,32].

Overfitting is a significant problem in machine-learning techniques. Reducing the number of inputs or parameters in models built with machine-learning algorithms is important for preventing overfitting. Eliminating irrelevant features from the dataset can notably improve the performance of machine-learning models, and feature importance is the method used to identify the most influential features for a given model within a dataset [33].

In machine-learning techniques, the number of inputs has been reduced by selecting from parameters used in different methods as inputs [34]. In this study, the Relief method was also used to select important parameters for each dataset. The five distinguishing features for classification for each of the three datasets (ICP-OES, XRF, and Vis-NIR) are presented in Table 5. Accordingly, for the ICP-OES dataset, elements such as CaO, Fe_2_O_3_, Al_2_O_3_, MgO, and MnO were selected by the Relief algorithm, while for the XRF dataset, SiO_2_, CaO, Fe_2_O_3_, Al_2_O_3_, and MnO were chosen. Particularly, concentration of elements such as SiO_2_, Fe_2_O_3_, Al_2_O_3_, and Ti can provide information about soils with varying degrees of weathering, shedding light on pedogenic processes and potential differences in parent materials [14]. In the Vis-NIR dataset, wavelengths 567, 571, 572, 573, and 574 nm were selected, with particular significance noted around 570 nm, potentially indicative of variations in soil color due to the presence of iron oxides. These wavelengths are also closely associated with soil texture and organic carbon content [35].

The results of the classifications performed using only the most effective features selected with Relief algorithms are presented in Table 6. In general, the utilization of Relief to diminish input parameters typically led to a slight decrease in accuracy or showed no significant difference when compared to models incorporating all variables. Although the classification accuracies were relatively less successful compared to models using all variables, they were at acceptable levels. The least influence occurred in the classification conducted using the XRF dataset (Table 6). Applying Relief to reduce the number of variables, except in EBT, has generally yielded more successful results for XRF compared to Vis-NIR in other machine-learning techniques. Especially in SVM, both in cross-validation and in the classification using an independent dataset, XRF’s accuracy rates were found to be higher than those of Vis-NIR (Table 6). This also demonstrated that reducing data in Vis-NIR in some cases could have a negative impact on the success results.

In machine-learning techniques, one of the best solutions to the overfitting problem encountered is evaluating models using cross-validation techniques [36]. Cross-validation is important for testing the robustness of models and evaluating their reliability. At a minimum, cross-validation should be conducted; however, a higher level of testing involves evaluating model performance with independent sample sets consisting of samples not used in model building, thus thoroughly assessing its performance. The ultimate goal is the model’s ability to be transferred to different domains and tested with samples obtained from areas outside the training set [34].

In this study, different types of validation strategies were tested: five out of cross-validation and splitting data into calibration and validation set, and, additionally, the opened soil profile samples were used in the classification of surrounding soil samples. Testing the model through cross-validation and random partitioning of the dataset to obtain independent sample sets yielded similar results. However, validations obtained by creating training sets from surface samples taken from opened profiles and presenting surrounding samples to the model resulted in relatively lower accuracy but promising outcomes. The relatively lower accuracy levels may be attributed to the low number of samples in the training set.

Among the compared classification algorithms, ESKNN generally yielded more successful results. When models were compared through cross-validation, the EBT technique was the most successful in the ICP-OES dataset, while ESKNN outperformed in models utilizing XRF and spectral data. In cases where models were tested with independent datasets, ESKNN produced the most successful results in models using XRF and spectral data, whereas SVM was found to be more successful in models using the ICP-OES dataset. Additionally, in models utilizing the variables determined by the Relief model, the algorithms’ performances remained similar to those of models using all variables, showing no significant changes in the models’ success rates.

The effectiveness of a classification algorithm often depends on the specific characteristics of the dataset being used, such as the nature of the data (e.g., imbalance, dimensionality, and presence of outliers) and the underlying relationships between variables. Each classification algorithm comes with its own set of advantages and disadvantages. Therefore, the choice of the “best” algorithm for a particular task often requires experimentation and evaluation to determine which one performs optimally given the specific data and objectives of the study [33].

While techniques like ICP and XRF offer superior accuracy in soil characterization compared to VNIRS, VNIRS can deliver rapid and cost-effective results with fewer sample-preparation requirements. Furthermore, both XRF and VNIRS techniques can also be used in the field, in addition to the laboratory, for the characterization of soils [14,37]. However, the accuracy of VNIRS in soil characterization, classification, and estimation may be influenced by soil mineral content. For instance, high soil organic matter or CaCO_3_ levels can reduce accuracy by limiting the contribution of other variables to the spectra. Specific spectral models adapted to the region and study area may also be necessary for an optimal performance [38,39].

## 4. Conclusions

This study aimed to accurately identify the sources of various soil samples taken from soil profiles opened at different parent materials and their surrounding areas for soil science purposes, utilizing advanced analytical techniques and classification approaches. ESKNN generally outperformed other classification algorithms. The inclusion of variables determined by the Relief model in models did not significantly alter the algorithms’ performances compared to models using all variables. The success of the models reaching up to 100% indicates the potential of the datasets and models in accurately distinguishing and determining the source of soils in soil science. Using samples from dug soil profiles to train the models and then testing them with surrounding samples resulted in lower accuracy, but it showed promise.

## Figures and Tables

**Table 1:** Some geographic information of parent material used in this study.

Profile No.	Number of Samples ^†^	Elevation (m)	Slope (%)	Soil Orders	Parent Material	Land Use
1	4	368	0–2	Typic Haploxerept	Mudflow	Irrigated agriculture
2	4	365	0–2	Fluventic Haploxerept	Mudflow	Irrigated agriculture
3	4	446	0–2	Aridic Haploxerert	Mudflow	Irrigated agriculture
4	6	644	0–2	Typic Haploxerept	Limestone	Field crops
5	7	599	0–2	Typic Haploxerept	Limestone	Field crops
6	6	586	2–4	Typic Haploxerept	Limestone	Semi-arid
7	5	658	2–4	Litic Haploxerept	Marn	Semi-arid
8	7	715	2–4	Typic Haploxerept	Marn	Pistachio
9	6	550	2–4	Typic Haploxerept	Marn	Semi-arid
10	7	1424	1–2	Typic Haploxerept	Basalt	Pasture
11	7	1031	2–4	Typic Haploxerept	Basalt	Pasture
12	6	612	0–2	Aridic Haploxerert	Basalt	Field crops

^†^ Number of samples included in soil classification, surface sample of each profile and samples taken from its surroundings.

**Table 2:** Working conditions of ICP-OES equipment.

Power	1350 watts
Plasma gas flow	15 L/min
Auxiliary gas flow	1.5 L/min
Nebulizer gas flow	0.75 L/min
Pump speed	15 rpm
Sample flow rate	1.5 mL/min

**Table 3:** The total element concentrations determined by ICP-OES and XRF techniques for different parent materials.

Total Elements	ICP–OES				XRF			
	Mudflow	Limestone	Marn	Basalt	Mudflow	Limestone	Marn	Basalt
SiO_2_ (%)	0.07 ± 0.05 ^a†^ (0.05–0.11)	0.07 ± 0.05 ^a^ (0.05–0.10)	0.09 ± 0.04 ^a^ (0.06–0.11)	0.10 ± 0.03 ^a^ (0.08–0.12)	42.57 ± 5.90 ^b^ (38.6–46.5)	52.32 ± 9.81 ^a^ (47.7–56.9)	24.87 ± 6.39 ^c^ (21.7–28.1)	56.4 ± 3.36 ^a^ (54.8–58.0)
Al_2_O_3_ (%)	2.98 ± 0.99 ^b^ (2.35–3.62)	4.48 ± 1.36 ^a^ (8.84–5.12)	2.37 ± 1.24 ^b^ (1.75–2.99)	5.04 ± 1.46 ^a^ (4.35–5.72)	8.72 ± 1.09 ^c^ (38.6–46.5)	12.14 ± 1.88 ^a^ (38.6–46.5)	5.72 ± 2.06 ^d^ (38.6–46.5)	10.95 ± 0.79 ^b^ (38.6–46.5)
CaO (%)	8.65 ± 3.29 ^b^ (6.56–10.75)	4.50 ± 5.00 ^c^ (2.16–6.84)	25.16 ± 9.39 ^a^ (20.49–29.8)	1.26 ± 1.22 ^c^ (0.68–1.83)	18.68 ± 4.29 ^b^ (15.8–21.6)	8.39 ± 8.63 ^c^ (4.4–12.4)	32.13 ± 8.75 ^a^ (27.8–36.5)	3.53 ± 1.60 ^d^ (2.8–4.3)
Fe_2_O_3_ (%)	2.81 ± 0.82 ^c^ (2.28–3.33)	4.16 ± 1.38 ^b^ (3.51–4.81)	2.63 ± 0.87 ^c^ (2.19–3.06)	5.29 ± 1.91 ^a^ (4.40–6.19)	5.46 ± 0.66 ^c^ (5.0–5.9)	7.59 ± 1.29 ^b^ (6.9–8.2)	3.78 ± 1.07 ^d^ (3.3–4.3)	11.43 ± 3.08 ^a^ (9.9–12.9)
MgO (%)	1.03 ± 0.30 ^b^ (0.84–1.22)	1.03 ± 0.28 ^b^ (0.89–1.16)	0.72 ± 0.38 ^c^ (0.53–0.91)	1.40 ± 0.28 ^a^ (1.26–1.53)	3.05 ± 0.17 ^a^ (2.9–3.2)	2.40 ± 0.41 ^b^ (2.2–2.5)	1.73 ± 0.65 ^c^ (1.4–2.1)	2.29 ± 0.42 ^b^ (2.1–2.4)
K_2_O (%)	0.23 ± 0.12 ^a^ (0.15–0.31)	0.25 ± 0.09 ^a^ (0.20–0.29)	0.16 ± 0.08 ^b^ (0.12–0.20)	0.20 ± 0.07 ^ab^ (0.17–0.23)	1.29 ± 0.29 ^a^ (1.1–1.5)	1.52 ± 0.26 ^a^ (1.4–1.6)	0.89 ± 0.27 ^b^ (0.7–1.0)	1.24 ± 0.70 ^a^ (0.1–1.6)
P_2_O_5_ (%)	0.003 ± 0.0014 ^b^ (0.002–0.004)	0.006 ± 0.002 ^a^ (0.005–0.007)	0.003 ± 0.0022 ^b^ (0.001–0.004)	0.006 ± 0.002 ^a^ (0.005–0.007)	0.15 ± 0.03 ^b^ (0.12–0.17)	0.17 ± 0.04 ^b^ (0.14–0.19)	0.17 ± 0.11 ^b^ (0.11–0.23)	0.37 ± 0.31 ^a^ (0.2–0.5)
MnO (%)	0.05 ± 0.02 ^b^ (0.04–0.07)	0.10 ± 0.04 ^a^ (0.08–1.12)	0.05 ± 0.02 ^b^ (0.03–0.06)	0.11 ± 0.04 ^a^ (0.09–0.13)	0.11 ± 0.02 ^b^ (0.09–0.12)	0.16 ± 0.04 ^a^ (0.14–0.18)	0.07 ± 0.02 ^c^ (0.06–0.08)	0.17 ± 0.04 ^a^ (0.15–0.20)

^†^ Statistically significant differences between groups with different letters (95%).

**Table 4:** Classification successes obtained through different classification techniques.

Classification Techniques	Validation Approach	Error Metrics	Datasets		
			ICP–OES	Vis-NIR	XRF
SVM	CV-5 ^†^	Acc.	0.77	0.97	0.91
		F_Score	0.67	0.99	0.95
		G_Mean	0.82	0.96	0.95
	Random partitioning ^‡^	Acc.	0.86	0.96	0.92
		F_Score	1	0.96	1
		G_Mean	1	0.96	1
	Profile-Based Training and Surrounding Validation Method ^β^	Acc.	0.76	0.80	0.74
		F_Score	0.70	0.67	0.63
		G_Mean	0.83	0.83	0.80
ESKNN	CV-5	Acc.	0.74	0.99	0.94
		F_Score	0.54	0.99	0.95
		G_Mean	0.69	0.99	0.95
	Random partitioning	Acc.	0.79	0.98	1
		F_Score	0.40	0.96	1
		G_Mean	0.62	0.99	1
	Profile-Based Training and Surrounding Validation Method	Acc.	0.64	0.76	0.65
		F_Score	0.67	0.67	0.70
		G_Mean	0.84	0.80	0.86
EBT	CV-5	Acc.	0.79	0.80	0.90
		F_Score	0.64	0.67	0.91
		G_Mean	0.78	0.76	0.94
	Random partitioning	Acc.	0.71	0.81	0.77
		F_Score	0.67	0.64	1
		G_Mean	0.71	0.76	1
	Profile-Based Training and Surrounding Validation Method	Acc.	0.57	0.69	0.62
		F_Score	0.58	0.58	0.58
		G_Mean	0.77	0.73	0.81

SVM, Support Vector Machine; ESKNN, Ensemble Subspace k-Near Neighbor; EBT, Ensemble Bagged Trees; ^†^ 5-fold cross-validation; ^‡^ the data were split into calibration (training 80% of the data) and validation sets (20% of the data); ^β^ using surface samples from opened soil profiles for training and validating the model with surrounding examples.

**Table 5:** Significant variables identified from each dataset using the Relief technique.

Datasets	The Most Effective 5 Features
ICP-OES	CaO, Fe_2_O_3_, Al_2_O_3_, MgO, and MnO
Vis-NIR	567, 572, 573, 571, and 574 nm
XRF	SiO_2_, CaO, Fe_2_O_3_, Al_2_O_3_, and MnO

**Table 6:** Classification successes obtained via different classification methods utilizing variables identified through Relief algorithms.

Classification Techniques	Validation Approach	Error Metrics	Datasets		
			ICP-OES	Vis-NIR	XRF
SVM	CV-5 ^†^	Acc.	0.70	0.71	0.88
		F_Score	0.60	0.48	0.63
		G_Mean	0.76	0.62	0.67
	Random partitioning ^‡^	Acc.	0.86	0.77	0.92
		F_Score	0.80	0.53	1
		G_Mean	0.94	0.63	1
	Profile-Based Training and Surrounding Validation Method ^β^	Acc.	0.65	0.76	0.62
		F_Score	0.63	0.63	0.53
		G_Mean	0.76	0.79	0.72
ESKNN	CV-5	Acc.	0.70	0.93	0.93
		F_Score	0.56	0.86	0.86
		G_Mean	0.74	0.90	0.89
	Random partitioning	Acc.	0.79	0.93	1
		F_Score	0.57	0.86	1
		G_Mean	0.82	0.95	1
	Profile-Based Training and Surrounding Validation Method	Acc.	0.53	0.74	0.59
		F_Score	0.54	0.64	0.60
		G_Mean	0.73	0.78	0.79
EBT	CV-5	Acc.	0.71	0.89	0.88
		F_Score	0.56	0.83	0.83
		G_Mean	0.67	0.89	0.92
	Random partitioning	Acc.	0.71	0.91	0.85
		F_Score	0.50	0.92	1
		G_Mean	0.66	0.97	1
	Profile-Based Training and Surrounding Validation Method	Acc.	0.52	0.66	0.53
		F_Score	0.56	0.51	0.58
		G_Mean	0.76	0.68	0.81

SVM, Support Vector Machine; ESKNN, Ensemble Subspace k-Near Neighbor; EBT, Ensemble Bagged Trees; ^†^ 5-fold cross-validation; ^‡^ the data were split into calibration (training 80% of the data) and validation sets (20% of the data); ^β^ using surface samples from opened soil profiles for training and validating the model with surrounding examples.

## Data Availability

Dataset available on request from the authors.
